# Liver Sinusoidal Endothelial Cell-Mediated CD8 T Cell Priming Depends on Co-Inhibitory Signal Integration over Time

**DOI:** 10.1371/journal.pone.0099574

**Published:** 2014-06-12

**Authors:** Julita Kaczmarek, Yahya Homsi, Jan van Üüm, Christina Metzger, Percy A. Knolle, Waldemar Kolanus, Thorsten Lang, Linda Diehl

**Affiliations:** 1 Institute of Molecular Medicine at the LIMES Institute, University of Bonn, Bonn, Germany; 2 Department of Membrane Biochemistry at the LIMES Institute, University of Bonn, Bonn, Germany; 3 Department of Molecular Immunology at the LIMES Institute, University of Bonn, Bonn, Germany; 4 Institute of Molecular Immunology, Technical University Munich, Munich, Germany; Centre de Recherche Public de la Santé (CRP-Santé), Luxembourg

## Abstract

The initiation of adaptive immunity requires cell-to-cell contact between T cells and antigen-presenting cells. Together with immediate TCR signal transduction, the formation of an immune synapse (IS) is one of the earliest events detected during T cell activation. Here, we show that interaction of liver sinusoidal endothelial cells (LSEC) with naive CD8 T cells, which induces CD8 T cells without immediate effector function, is characterized by a multi-focal type IS. The co-inhibitory molecule B7H1, which is pivotal for the development of non-responsive LSEC-primed T cells, did not alter IS structure or TCRβ/CD11a cluster size or density, indicating that IS form does not determine the outcome of LSEC-mediated T cell activation. Instead, PD-1 signaling during CD8 T cell priming by LSEC repressed IL-2 production as well as sustained CD25 expression. When acting during the first 24 h of LSEC/CD8 T cell interaction, CD28 co-stimulation inhibited the induction of non-responsive LSEC-primed T cells. However, after more than 36 h of PD-1 signaling, CD28 co-stimulation failed to rescue effector function in LSEC-primed T cells. Together, these data show that during LSEC-mediated T cell priming, integration of co-inhibitory PD-1 signaling over time turns on a program for CD8 T cell development, that cannot be overturned by co-stimulatory signals.

## Introduction

The initiation of adaptive immunity is dependent on the physical interaction of an antigen-presenting cell (APC) with a naïve T cell. This results in the formation of an immune synapse (IS), in which the T cell receptor (TCR) rearranges to form a highly organized central supra-molecular activation cluster (c-SMAC) [1], surrounded by adhesion molecules like CD54 in the peripheral SMAC (p-SMAC). IS formation is initiated by TCR signaling and is maintained via the constant centripetal translocation of TCR micro-clusters, with associated signaling molecules, from the periphery into the c-SMAC, where signaling molecules dissociate [2]. Additionally, in recent years, multi-focal synapses and kinapses, in which T cells can acquire and integrate signals whilst migrating [3], have been described. Although T cells can form all three types of synapses depending on the type of APC they encounter [4] it is not clear whether the type of immune synapse correlates with the outcome of the immune response that is initiated by this interaction.

The mechanisms governing the regulation of innate and adaptive immune responses are many-fold, and include the induction of regulatory cells and/or cytokines. In the liver, sinusoidal endothelial cells (LSEC), an organ-resident APC population, can add to this regulation [5] via interaction with CD4 and CD8 T cells, which leads to the development of regulatory functions in CD4 [6], [7] and the B7H1/PD-1-mediated silencing of immediate effector function in CD8 T cells [8], instead CD8 T cells survive and can develop into memory cells with anti-infectious activity [9].

Here, we investigate at the level of the immune synapse the interaction of wild type and B7H1-deficient LSEC with naïve CD8 T cells leading to T cell non-functionality or T cell activation. We addressed the question whether the form of the immune synapse parallels the functional outcome of CD8 T cell priming. Our data show that multifocal immune synapses characterize the interaction between antigen-presenting LSEC and naïve CD8 T cells. However, B7H1/PD-1 signaling, which is essential for the induction of LSEC-primed CD8 T cells that lack immediate effector function, did neither alter IS form, nor influence the cluster size or density of the TCR and CD11a. In contrast, we found that CD8 T cells primed by LSEC required B7H1-dependent signal integration for more than 36 h in order to acquire the particular differentiation state of non-functionality, which after this time point was not reversible any more by co-stimulatory signals delivered through CD28. Thus, LSEC can induce a B7H1-dependent non-functional state in CD8 T cells, which does not depend on a particular immune synapse phenotype, but rather requires integration of co-inhibitory PD-1 signaling over a longer period of time.

## Materials and Methods

### Mice for isolation of LSEC and T cells

C57BL/6J, B7H1-/-, H-2K^b^SIINFEKL-restricted TCR-transgenic (OT-1), OT-1×PD-1^-/-^ and H-2K^b^-restricted DesTCR mice were bred in the central animal facility in Bonn according to the Federation of European Laboratory Animal Science Association guidelines and maintained under SPF conditions. All efforts were taken to minimize suffering. Mice were not subjected to any injections or manipulation before sacrifice by cervical dislocation. Then organs were taken for isolation of LSEC from liver or T cells from spleen. This is not classified as an animal experiment by the Animal Care Commission of Nordrhein-Westfalen and requires notification but not approval.

### Coculture experiments

LSEC were isolated from livers as described [8]. LSEC were used 2–3 days after preparation and were routinely 95–100% confluent. B6 or B7H1^-/-^ LSEC were cultured on collagen-coated 24-well or 96-well plates and co-cultured with 10^6^ or 10^5^ DesTCR CD8 T cells, OT-1 CD8 T cells or PD-1^-/-^ OT-1 T cells. OT-1 containing cultures were performed in the presence of 100 µg/ml OVA. After the indicated time-points 5 µg/ml anti-CD28 (37.51, eBisocience) or isotype control antibody was added to the coculture. 4 days later cells were harvested and restimulated with PMA (5 ng/ml, Sigma Aldrich) and Ionomycin (200 ng/ml, Sigma) for 4 h in presence of Brefeldin A and Monensin (eBioscience), after which they were stained intracellularly for IL-2/IFNγ or supernatants were used for IL-2/IFNγ Elisa, respectively.

### Immunofluorescent staining

LSEC were grown on collagen-coated coverslips and loaded with 0,1 mg/ml OVA (for coculture with OT-1 T cells) or left untreated (for coculture with DesTCR T cells) for 1–2 hours. To ensure that the start of LSEC/T cell interaction was synchronized naïve T cells were centrifuged onto the LSEC for 1′ at 1000 rpm. For TIRF microscopy cells were fixed after the indicated time-points in 4% paraformaldehyde, blocked with Tris-Buffered Saline containing 1% BSA/1% donkey serum (Jackson Immunoreasearch) and stained with anti-TCRβ (H57-597, eBioscience), Alexafluor-488 Goat-anti-Hamster IgG (H+L, Molecular Probes) as secondary antibodies or anti-CD11a (I21/7, Southern Biotech) antibodies, Alexafluor-488 Goat-anti-Rat IgG (H+L, Molecular Probes) as secondary antibodies. After washing coverslips were mounted in ProlongGold (Invitrogen), supplemented with 50 µg/mL DABCO anti-fade reagent (Sigma-Aldrich), and analyzed. For confocal microscopy cells were incubated with avidin/biotin blocking agent (Invitrogen) and stained with biotinylated anti-TCRβ and unlabeled anti-CD11a antibodies and Cy5-labeled streptavidin and Cy3-labeled anti-Rat-IgG (Jackson Immunoresearch).

### Western blot

Cell were lysed in lysis buffer (20 mM tris-HCl pH 7.5, 150 mM NaCl, 1 mM EGTA, 1 mM EDTA, 2.5 mM sodium pyrophosphate, 1 mM β-glycerophosphate, 1 mM sodium vanadate, 1% v/v Triton X-100) and extracts were separated by 9–12% SDS-PAGE. Proteins were electrotransferred onto PVDF membranes. Immunoblots used antibody solutions in 5% BSA in TBS (10 mM Tris-HCl, pH 8.0, and 150 mM NaCl) and washes used TBS containing 0,1% Tween-20. Relative band intensities were quantified using ImageJ. The following antibodies were used: anti-pCD3ζ (clone K25-407.69, BD Biosciences), anti-CD3ζ (Proteintech Europe), anti-pLck, Lck (cat.no 2751 and 2752 from Cell Signaling Technologies) and anti-β-actin (Santa Cruz Biotechnology).

### Total internal reflection (TIRF) and confocal microscopy

Confocal microscopy was performed using an Olympus Fluoview 1000 confocal microscope equipped with a Plan Apochromat 60×, NA 1.4 oil immersion objective (Olympus). TIRF microscopy was performed as described previously [10]. For image autocorrelation analysis, we used the ImageJ program. Squared regions of interest (ROIs) with a size of 45 pixel×45 pixel were placed within the contact site and the ROI was correlated with the original image (yielding a correlation coefficient of 1). Then the original image was displaced pixel-wise up to 7 pixels and the correlation coefficient was determined after each displacement. The operation was performed in all four directions (up, down, right, left) and the four values were averaged yielding an autocorrelation curve for the respective ROI. Autocorrelation curves from individual cells were averaged for one independent experiment. Values are given as mean ± SD (n = 5 cells).

### Statistics

All experiments were performed at least three times with groups of 3 mice unless otherwise stated. Results are expressed as mean ± SEM. Statistical significance was calculated using ANOVA or Student *t*-test (*p≤0.05, **p≤0.01, ***p≤0.001).

## Results

### Contact area between antigen-presenting LSEC and naïve CD8 T cells recognizing their cognate antigen resembles a multifocal immune synapse

**Figure 1:**
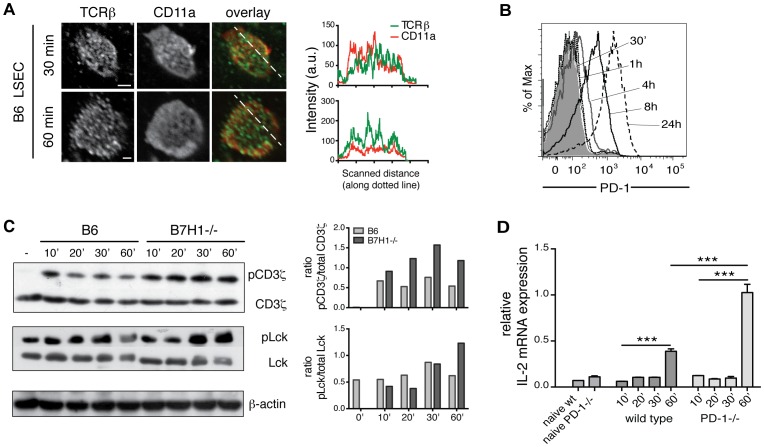
Naïve CD8 T cell/LSEC interaction resembles a multifocal synapse. **A**: OVA-loaded B6 were cultured with naïve OT-1 CD8 T cells and after the indicated times cells were fixed and stained for TCRβ (green) and the LFA-1 subunit CD11a (red) and analyzed by confocal microscopy. Line-scans show signal intensities (arbitrary units) along the dotted lines for TCRβ (green) and CD11a (red) in the overlay. Scale bar shows 10 µm. Representative data from 3 independent experiments are shown. Images are shown at arbitrary scaling. **B**: Naïve OT-1 T cells were co-cultured with B6 LSEC with or without OVA for the indicated times and stained for CD8 and PD-1. The histogram shows PD-1 expression over time gated on viable CD8 T cells. Filled grey histogram represents isotype control staining. **C**: OT-1 CD8 T cells were cultured with antigen-presenting wild type or B7H1-/- LSEC for the indicated times after which cell lysates were probed for protein expression by western blot as indicated and quantified. **D**: Wild type or PD-1^-/-^ OT-1 CD8 T cells were cultured with antigen-presenting LSEC for the indicated times after which IL-2 mRNA levels were determined by real time PCR. Representative data from at least 3 independent experiments are shown. Data are shown as mean +/- SEM. Significance was calculated by ANOVA. *p≤0.05, **p≤0.01, ***p≤0.001.

The interaction between T cells and antigen-presenting cells results in the rapid formation of a highly organized structure in which TCR micro-clusters form a central SMAC (cSMAC), which is surrounded by a peripheral SMAC (pSMAC) enriched in adhesion molecules like LFA-1 [1]. However, apart from this classical bull's eye immune synapse more recently other forms, such as a multifocal synapse or kinapse have been described [4]. To investigate what type of interaction characterizes the interaction between naïve CD8 T cells and LSEC, we co-cultured naïve CD8 OT-1 T cells with OVA-loaded LSEC for 30′ or 60′, fixed and stained the cells with the cSMAC-associated TCRβ and the pSMAC-associated LFA-1 subunit CD11a. T cells established contacts to extremely flat glass attached LSEC, allowing imaging of the contact site within a single confocal x,y-scan. Confocal micrographs from the LSEC-T-cell contact site revealed that TCRβ and CD11a clusters are largely non-overlapping in the membrane of naïve OT-1 T cells interacting with antigen-presenting LSEC (**Fig. 1A**). These clusters did not form a ring-structure typical of a bull's eye synapse. Instead the antigen-specific interaction between naïve CD8 T cells and LSEC led to a structure resembling a multifocal immune synapse.

### B7H1/PD-1 signaling rapidly interferes with T cell signal transduction strength

We have previously described that naïve CD8 T cells stimulated by LSEC develop into a non-functional state, in which they are unable to perform direct cytotoxic function or produce cytokines upon restimulation via the TCR. The development of this non-functional state depends on LSEC-expressed B7H1 [8]. Flow cytometric analysis of the kinetics of PD-1 expression on T cells induced by LSEC revealed that PD-1 protein was antigen-dependently upregulated between 1 h and 4 h (**Fig. 1B** **, Fig. S1**). Moreover, the absence of PD-1/B7H1 dependent signaling led to enhanced proximal TCR signal transduction (enhanced phosphorylation of CD3ζ and Lck) as soon as 30′ to 60′ after T cell activation (**Fig. 1C**). PD-1 signaling has been shown to inhibit IL-2 production in T cells [11] and we found that as soon as 1 h after stimulation IL-2 mRNA induction in naïve PD-1^-/-^ CD8 T cells cultured with antigen-presenting LSEC was significantly increased as compared to wild type CD8 T cells (**Fig. 1D**), indicating that PD-1 dependent signals are rapidly translated into a differential response as early as 30-60′ after antigenic stimulation by LSEC

### LSEC-mediated B7H1-signals do not affect TCRβ and CD11a cluster size or density

**Figure 2:**
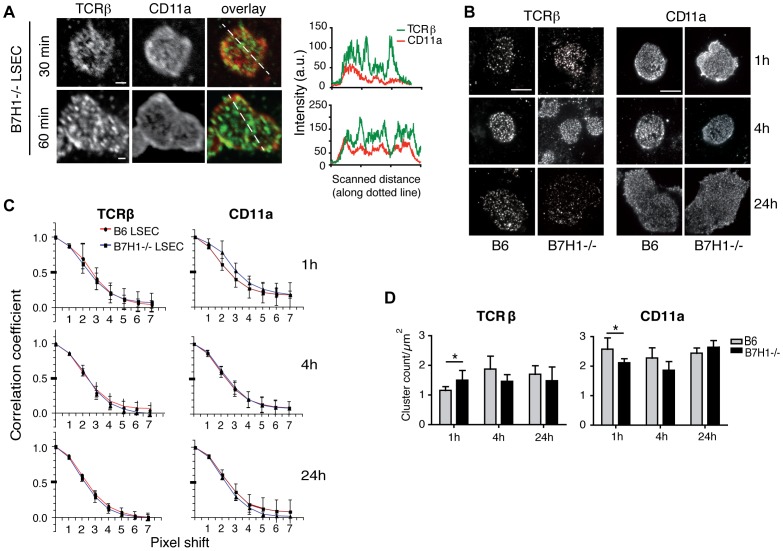
TCRβ and CD11a distribution in naïve CD8 T cell/LSEC interaction is not affected by B7H1-dependent signals. **A**: OVA-loaded B7H1^-/-^ LSEC were cultured and stained as in Fig. 1A and analyzed by confocal microscopy. Scale bar shows 10 µm. Images are shown at arbitrary scaling. Representative data from 3 independent experiments are shown. **B**: OVA-loaded B6 and B7H1^-/-^ LSEC were cultured with naïve OT-1 CD8 T cells and after the indicated times cells were fixed and stained for TCRβ and CD11a and analyzed by TIRF microscopy. Scale bar shows 6 µm. **C**: TCRβ and CD11a cluster sizes were examined by autocorrelation analysis: the approximate average half object size is proportional to the pixel shift leading to a correlation coefficient of 0.5 (n = 5 cells; values are given as mean ± SD; one pixel corresponds to 83.3 nm). **D**: Clusters were counted within a 14,051 µm^2^ area/T cell and cluster density is given as clusters per µm^2^ (n = 5). Shown is the mean +/- SD. Representative data from 3 independent experiments. Data are shown as mean +/- SEM. Significance was calculated by ANOVA. *p≤0.05, **p≤0.01, ***p≤0.001.

As changes in signal strength can lead to changes in immune synapse cluster characteristics in T cells [12], [13], we then investigated whether the lack of inhibitory B7H1 signaling by LSEC would influence and/or change the development of a multifocal synapse. However, using B7H1^-/-^ LSEC for coculture with naïve CD8 T cells, we found that the lack of B7H1 signaling did not prevent the formation of a multifocal type of immune synapse (**Fig. 2A**) by confocal microscopy. We aimed for a more detailed quantitative analysis and investigated whether the size and density of the TCRβ and CD11a clusters within the interaction plane between LSEC and T cells was altered due to the lack of B7H1-dependent signaling by LSEC. To visualize single TCRβ or CD11a protein clusters in the T cell membrane, we used total internal reflection (TIRF) microscopy. LSEC are at the T-cell-LSEC contact very thin, and immunostained TCRβ and CD11a clusters in the membrane of naïve CD8 T cells can be excited by an evanescent wave that penetrates the LSEC (**Fig. 2B**). Again, we observed single TCRβ and CD11a clusters that were not spatially organized like a bull's eye synapse, confirming the data obtained by confocal microscopy. When we analyzed the average size distribution of TCRβ and CD11a clusters by autocorrelation analysis (**Fig. 2C**), we found no changes between cluster size in T cell membranes interacting with B6 or B7H1-deficient LSEC, nor did we find evidence for a change in cluster densities in the T cell membrane upon interaction with LSEC (**Fig. 2D**) at early (1h) or late (24 h) time points. Together, these data indicate that although T cell stimulation by B6 or B7H1^-/-^ LSEC leads to distinct functional outcomes, i.e. a change from non-responsive to activated IL-2 secreting T cells, such differential signaling did not correlate with the phenotype of the immune synapse during early T cell stimulation by antigen-presenting LSEC.

### B7H1/PD-1 signaling represses IL-2 production by LSEC-stimulated CD8 T cells

**Figure 3:**
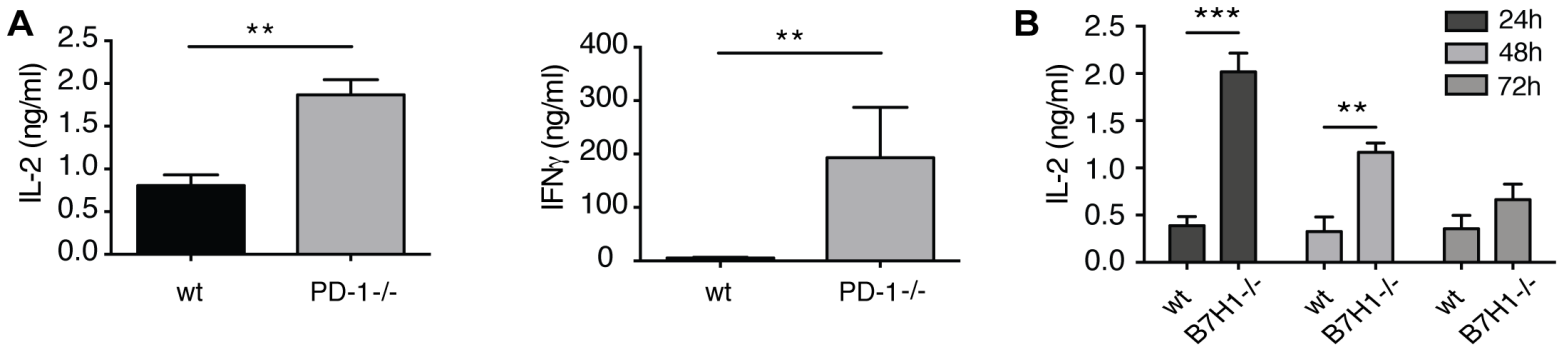
B7H1/PD-1 signaling suppresses IL-2 production in LSEC-primed T cells. **A**: Wild type or PD-1^-/-^ OT-1 CD8 T cells were cultured with B6 LSEC for 4 days and restimulated with plate-bound anti-CD3ε antibodies. After 24 h IL-2 and IFNγ content in the supernatant was determined by Elisa. **B**: OT-1 CD8 T cells were cultured with antigen-presenting LSEC from B6 or B7H1^-/-^ mice for the indicated times and the IL-2 concentration in the supernatant was determined by Elisa. Data are depicted as mean +/- SEM. Data are representative from 3 independent experiments. Significance was calculated by ANOVA. *p≤0.05, **p≤0.01, ***p≤0.001.

It has been proposed that full CD8 T cell activation is dependent on the integration of signaling over time [14], which results in sustained early IL-2 production and CD25 expression in CD8 T cells that are to become fully activated. We first confirmed that PD-1 expression on CD8 T cells, similar to B7H1 on LSEC, is pivotal for LSEC-induced CD8 T cell non-responsiveness (**Fig. 3A**). This was preceded by an increased production of IL-2 protein by CD8 T cells when cocultured with B7H1^-/-^ LSEC (**Fig. 3B**). Thus, our data suggest that LSEC utilize the regulatory B7H1/PD-1 signaling pathway to dampen early IL-2 production by T cells preventing their full activation.

### Co-stimulatory CD28 signaling cannot prevent LSEC-induced T cell non-functionality after prolonged co-inhibitory B7H1/PD-1 signaling

**Figure 4:**
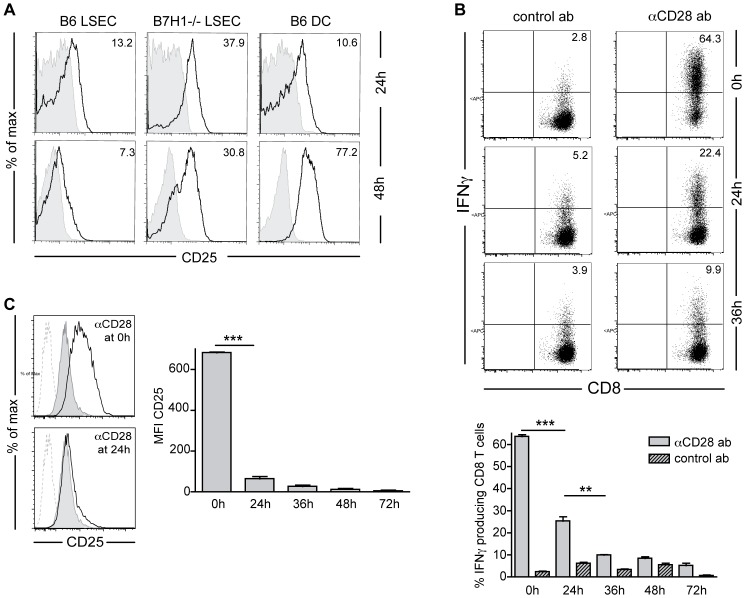
CD28 co-stimulation cannot reverse the induction of LSEC-primed T cells after PD-1 signal integration time. **A**: OT-1 CD8 T cells were co-cultured with antigen-presenting B6, B7H1^-/-^ LSEC or B6 DC for the indicated times and stained for CD8 and CD25. Grey filled lines: isotype control, black lines: CD25. Histograms show viable CD8 T cells. Numbers indicate geometric mean of CD25. **B**: OT-1 CD8 T cells were co-cultured with antigen-presenting LSEC for 4 days, after which they were restimulated with PMA/ionomycin and 4 h later stained for CD8 and IFNγ. Anti-CD28 antibodies (10 µg/ml) or isotype control antibodies were added to the co-cultures at the indicated times. Bar graph shows percentages of IFNγ-producing CD8 T cells upon restimulation at day 4 after CD28 abs or control abs were added at the indicated times. **C**: OT-1 CD8 T cells were co-cultured with antigen-presenting LSEC for 4 days, after which they were stained for CD8 and CD25. Anti-CD28 antibodies (5 µg/ml) or isotype control antibodies were added to the co-cultures at the indicated times. Histograms show CD25 expression on viable CD8 T cells, black line: with anti-CD28; filled grey: with control antibody; dotted: unstained. Bar graph shows CD25 mean fluorescence intensity at day 4 on viable CD8 T cells co-cultured with LSEC and anti-CD28 antibodies added at the indicated times. Representative data of 3 independent experiments are shown. Data are depicted as mean +/- SEM. Significance was calculated by ANOVA. *p≤0.05, **p≤0.01, ***p≤0.001.

CD8 T cells stimulated by antigen-presenting LSEC upregulate activation markers and proliferate to the same extent as DC-stimulated T cells. However, LSEC-primed T cells do not sustain activation marker expression, but reverse to a CD25^neg^CD62L^high^ phenotype [8]. PD-1 expression on LSEC-primed T cells reached its maximum after 24–48 h (**Fig. 1B**) and was retained for at least 5 days (data not shown), indicating that CD8 T cells remained receptive to co-inhibitory signaling during this whole time period. In the presence of B7H1 signaling CD25 down-regulation on LSEC-primed T cells was completed after 48 h (**Fig. 4A**). Therefore, we wondered whether not only activation of CD8 T cells requires signal integration over time [14] but also LSEC-mediated attenuation of CD8 T cell function. To investigate this, we added co-stimulatory anti-CD28 antibodies at different time-points into co-cultures of LSEC and CD8 T cells. As we have reported previously, when added during the beginning of coculture this induced full T cell activation as measured by IFNγ production upon restimulation ([8] and **Fig. 4B** upper panels). However, if we allowed for 24 h of B7H1/PD-1 signaling in CD8 T cells to occur during stimulation by antigen-presenting LSEC before adding agonistic co-stimulatory CD28 antibodies, we found that PD-1 signal integration into CD8 T cells sufficed to dampen their IFNγ production upon subsequent restimulation. After 36 h of PD-1 signal integration CD28-mediated co-stimulation failed to significantly increase IFNγ production above background levels altogether (**Fig. 4B**). The ability of the LSEC-primed CD8 T cells to produce IFNγ when CD28-stimulation was delivered early during the coculture correlated with the sustained expression of CD25 by these T cells (**Fig. 4C**). Thus, these data show that B7H1/PD-1 signals are integrated over a period of approximately 24 to 36 h during coculture with antigen-presenting LSEC. After this time co-stimulatory signals via CD28 to CD8 T cells cannot overcome the differentiation program that results in an activation refractory state. Taken together, these results indicate that the development of the unique differentiation state of LSEC-stimulated CD8 T cells is not merely due to a lack of co-stimulation, but involves active inhibitory signaling that needs to be integrated over time and that this unique differentiation process does not correlate with a particular composition of the immune synapse during these time points.

## Discussion

In the study presented here, we explored the characteristics of the immune synapse formed between antigen-presenting LSEC and CD8 T cells, that undergo a particular differentiation program that renders them unable to perform immediate effector function upon reactivation via the TCR [8], [15]. The first reports on immune synapses showed that upon contact with MHC- and CD54-loaded lipid bilayers T cells formed a large cluster of TCR, the central-SMAC, surrounded by a peripheral SMAC composed of adhesion molecules [16]. More recently, multifocal synapses [17], [18] and kinapses [3] have broadened the spectrum of immune synapse forms. Directed secretion of mediators, like perforin or granzyme by CTL [19], is observed in classical immune synapses, whereas kinapses are rather formed in migrating T cells. We made use of the particular thin and extended form of LSEC that allows imaging of immune synapse formation at high resolution during the natural interaction of an APC with naïve CD8 T cells. As naïve CD8 T cells stop migrating after MHC I-restricted recognition of antigen on LSEC [20], we did not detect kinapse formation, as expected. Instead, we found that during priming of naïve CD8 T cells by LSEC a multifocal immune synapse was formed, in which we observed both TCRβ and CD11a clusters by confocal microscopy in the contact area. However, no overlap or spatial segregation into a c- or p-SMAC of TCRβ and CD11a protein clusters were observed. Moreover, although PD-1 can be recruited into immune synapses [13] and modifies proximal TCR signaling strength [21], PD-1 signaling in naïve CD8 T cells undergoing priming by LSEC did neither affect immune synapse form nor the size or density of individual TCRβ and CD11a clusters within the synapse. As PD-1 signaling is pivotal for development of the non-responsive phenotype in LSEC-primed T cells we conclude from these results that the dynamics of immune synapse formation does not contribute to the distinct programming of T cell differentiation by antigen-presenting LSEC.

Naïve CD8 T cells upregulate PD-1 after activation via the TCR. Although there was no detectable increase in PD-1 protein expression levels on the cell surface of CD8 T cells after 60 minutes of co-incubation with antigen-presenting LSEC, PD-1 mediated signaling controlled the production of IL-2 mRNA at this early time point after TCR stimulation. PD-1 protein expression levels then increased within 4 h until reaching a maximum at 24–48 h and then remained stable for at least 4 days. As LSEC selectively upregulate co-inhibitory B7H1, but not co-stimulatory CD80 or CD86, during antigen-specific interaction with CD8 T cells [8], this implies that T cells continuously receive high levels of co-inhibitory, but insufficient co-stimulatory, signals during contact with antigen-presenting LSEC. Although LSEC do express other co-stimulatory molecules, like ICOSL (data not shown) and CD40 [22], the presence of these molecules does not overcome B7H1-dependent inactivation of LSEC-stimulated CD8 T cells.

For the development into fully functional effector T cells, naïve T cells need to receive sustained TCR signaling for a distinct period of time [14]. Naïve T cells that are given only a brief TCR stimulus, only transiently express CD25 and do not develop into effector T cells [14]. Similarly, CD8 T cells primed by LSEC also only transiently expressed CD25 as a consequence of co-inhibitory B7H1/PD-1 signaling. Augmenting the amount of IL-2 present in the LSEC/CD8 T cell co-cultures, either by adding exogenous IL-2 or inducing its production via agonistic anti-CD28 antibodies can effectively prevent the development of LSEC primed non-responsive CD8 T cells [8], [23]. Together, this suggests that LSEC-expressed B7H1 represses IL-2 production in CD8 T cells that is necessary to induce and sustain expression of CD25. Indeed, like LSEC-primed T cells, IL2-deficient CD8 T cells, that are unable to provide autocrine IL-2 protein, are impaired in their ability to respond to a second antigenic challenge [24].

Our data further show that not only T cell activation requires integration of stimulatory signal over time, but also the development of the unique differentiation state of LSEC-primed T cells depends on integration of co-inhibitory signals over time. CD28 co-stimulatory signaling was not able to induce full T cell priming anymore after 36 h of PD-1 signal integration during contact with antigen-presenting LSEC. Thus, the key events in LSEC-induced T cell differentiation occur during the first 24 to 36 hrs of cell-cell contact and are not reflected in a particular form or size of the immune synapse.

The main mechanism by which PD-1 signaling inhibits IL-2 production in T cells is by interfering with PI3K activation [25]. Upon T cell activation, PI3K activity can be induced via the TCR directly and augmented considerably via CD28- and/or CD25-mediated signals [26]. During CD8 T cell priming by LSEC inhibition of CD25-induced PI3K activity is the most relevant, as LSEC do not provide co-stimulation through CD28. Indeed, when activated CD8 T cells are stimulated with IL-2 in the presence of PI3K inhibitors, these cells do not develop further into effector cells, but return to being CD62L^high^, CCR7^pos^ T cells that home to secondary lymphoid organs [27], which is highly reminiscent of LSEC-primed T cells [9]. This key role of PD-1 in the unique T cell differentiation by antigen-presenting LSEC is consistent with the absence of any particular changes in immune synapse formation observed by us as PD-1-mediated co-inhibition interferes downstream of membrane-proximal TCR signals.

In summary, our study reveals that CD8 T cells recognizing antigens presented by LSEC form a multifocal immune synapse with similar TCRβ and CD11a characteristics, irrespective of whether those T cells are activated or rendered non-responsive. Signals originating from the B7H1-PD-1 axis are pivotal for the induction of the unique differentiation state of LSEC-primed T cells. LSEC-primed T cells integrate TCR and co-inhibitory PD-1 signals over a period of 24-36h after which this particular differentiation program cannot be reversed anymore by co-stimulatory signaling. Collectively, our data provide first evidence that distinct T cell differentiation processes are not associated with particular forms of immune synapse or size of immune synapse clusters but rather by integration of signaling downstream of the TCR.

## Supporting Information

Figure S1 PD-1 expression kinetics on LSEC-primed CD8 T cells. Naïve OT-1 T cells were cocultured with LSEC in the presence or absence of antigen for the indicated times. Bar graph depicts mean fluorescence intensity of PD-1 expression (n = 3).
(TIF)
