# Supplementary figures and images for: Liver Sinusoidal Endothelial Cell-Mediated CD8 T Cell Priming Depends on Co-Inhibitory Signal Integration over Time
Source: PLoS One. 2014 Jun 12;9(6):e99574. doi: 10.1371/journal.pone.0099574 (PMC4055751; doi:10.1371/journal.pone.0099574)

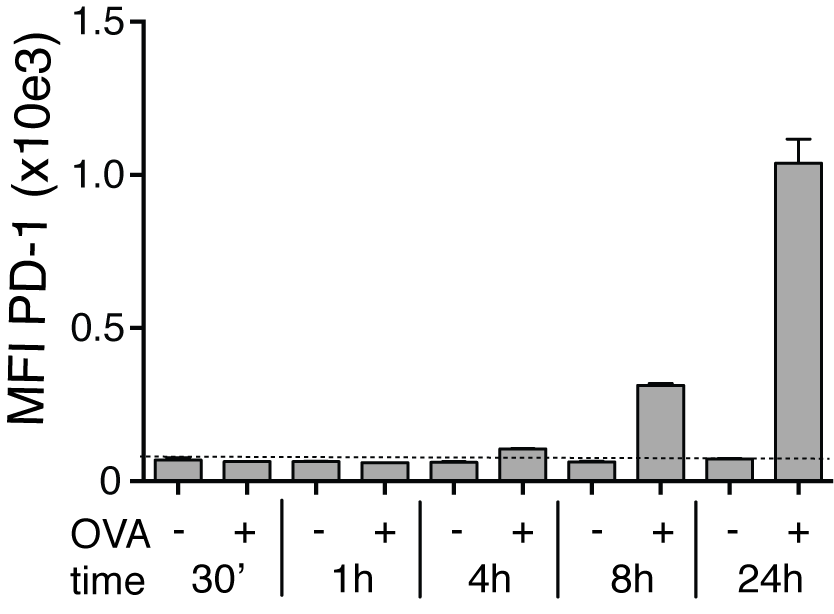

Supplement: Figure S1 — PD-1 expression kinetics on LSEC-primed CD8 T cells. Naïve OT-1 T cells were cocultured with LSEC in the presence or absence of antigen for the indicated times. Bar graph depicts mean fluorescence intensity of PD-1 expression (n = 3). (TIF) [file pone.0099574.s001.tif]
